# Hormone-dependent immune regulation shapes asthma heterogeneity across the female lifespan

**DOI:** 10.3389/fimmu.2026.1864024

**Published:** 2026-07-20

**Authors:** Qiaoyu Yang, Shucheng Hua

**Affiliations:** Department of Resperatory Medicine, The First Hospital of Jilin University, Changchun, China

**Keywords:** asthma, estrogen, female, hormone, immunity, inflammation, sex difference

## Abstract

Asthma exhibits pronounced sex differences across the human lifespan, shifting from a predominance in males during childhood toward an increased prevalence, severity, and heterogeneity in females following puberty. These epidemiological patterns are not paralleled by uniform changes in disease phenotype, with adult females exhibiting a higher prevalence of non-type 2 inflammation. Mechanistically, estrogen has emerged as a key regulator of this heterogeneity through its pleiotropic effects on immune and structural cells. However, the immunological actions of estrogen are highly context-dependent, varying with receptor subtype (ERα versus ERβ), hormone concentration, and prevailing inflammatory milieu. Rather than uniformly amplifying immunity, estrogen’s principal nuclear receptors exert opposing effects. ERα predominantly promotes immune activation, whereas ERβ exerts counterbalancing anti-inflammatory and bronchoprotective functions. Estrogen promotes immune amplification rather than polarization by simultaneously disrupting regulatory pathways and enhancing T helper 2 (Th2)-, T helper 17 (Th17)-, and innate immune system-mediated responses. Sex chromosome-linked and epigenetic mechanisms, including X-chromosome inactivation escape, further contribute to immune activation in females, although direct evidence in the asthma-specific context remains limited. Importantly, asthma phenotypes are shaped across the female lifespan by interactions between hormonal signaling and other contributing factors such as obesity and other comorbidities, viral infection, and environmental pollutant exposure, as well as emerging non-type 2 pathways including NLRP3 inflammasome activation and neutrophil extracellular trap formation, leading to context-dependent inflammatory patterns. This review summarizes the role of estrogen receptors and various cell types in mediating asthma heterogeneity, providing a mechanistic basis for sex-specific disease variation and highlighting the need for precision strategies that account for hormonal and life-stage differences.

## 1 Introduction

Asthma, a chronic inflammatory airway disease characterized by airway inflammation, bronchial hyperresponsiveness, and structural remodeling of the airway wall (1–5), exhibits marked sex-related differences in prevalence and clinical severity. During childhood, asthma occurs more frequently in boys than in girls; however, this pattern reverses after puberty, with the prevalence and severity being higher in adult females than in adult males (6–9). These observations strongly suggest that biological sex and sex hormones contribute to asthma heterogeneity. This review summarizes the impact of estrogen and various immune and inflammatory cell types on asthma phenotypes in females across the lifespan.

## 2 Estrogen receptors and signaling in asthma

Estrogen encompasses structurally related steroid hormones that differ in receptor-binding affinity and immunological potency. 17β-estradiol (E2) is the predominant circulating estrogen during the reproductive years and exhibits the highest affinity for both ERα and Erβ. E2 is the primary form studied in most experimental and clinical contexts cited in this review. Estriol (E3) is markedly elevated during pregnancy and produced by the fetoplacental unit. The binding affinity of E3 for ERα and ERβ is approximately 11–14% and 17–21% that of E2, respectively, and displays a preference for ERβ over ERα, different to E2 (10, 11). Following menopause, circulating E2 declines markedly and is replaced by estrone (E1) as the predominant endogenous estrogen. E1 is derived primarily from the peripheral aromatization of adrenal androstenedione rather than direct ovarian secretion, and it activates ERα and ERβ with substantially lower potency than E2, which may partly explain why menopausal data do not linearly extrapolate from reproductive-age findings (12). In addition, locally synthesized estrogens in airway epithelial and stromal cells may act in an autocrine or paracrine manner (13). Finally, environmental endocrine-disrupting chemicals, including bisphenol A and phthalates, can activate estrogen receptors and are discussed separately in Section 6 in the context of environmental pollutant exposure. These distinctions are important because the immunological effects described throughout this review are predominantly established for E2, and these concepts should be generalized to other molecular with caution.

Estrogen regulates both innate and adaptive immune responses through multiple receptor-mediated mechanisms. Several immune cell populations that have been implicated in the pathogenesis of asthma express estrogen receptors, including group 2 innate lymphoid cells (ILC2s), eosinophils, mast cells, and T lymphocytes. The principal estrogen receptors involved in immune regulation include estrogen receptor alpha (ERα), estrogen receptor beta (ERβ), and the G protein-coupled estrogen receptor (GPER, also known as GPR30) (14, 15), with the diverse immunomodulatory effects of estrogen being predominantly mediated through ERα rather than ERβ. In addition to classical nuclear receptor signaling, estrogen can also activate rapid non-genomic pathways through GPER, a membrane-associated receptor that can be directly engaged to initiate intracellular signaling cascades. Activation of each type of receptor initiates distinct intracellular pathways that regulate immune cell differentiation, survival, and effector functions (16, 17). In addition to its immunomodulatory effects, estrogen can also influence airway structural cells, potentially by altering epithelial barrier integrity, reducing airway surface liquid height, and promoting ferroptosis in airway epithelial cells; all of these processes have been implicated in airway hyperresponsiveness and airway remodeling (18).

Beyond receptor-mediated signaling, estrogen may also interact with sex chromosome-linked regulatory mechanisms. Many immune-related genes are located on the X chromosome, and their expression is tightly regulated through X-chromosome inactivation (XCI), which silences one of the two X chromosomes in female cells to achieve dosage compensation with males. XCI is initiated by X-inactive specific transcript ribonucleic acid-mediated chromatin remodeling, leading to stable transcriptional repression of most X-linked genes. However, a subset of genes escapes such inactivation to remain biallelically expressed, resulting in higher expression levels in females than in males. Several immune-related genes are among these escapees, providing a potential mechanism underlying the observed sex differences in immune responses. Estrogen signaling may influence the transcriptional regulation of these X-linked genes, thereby contributing to sex-specific immune responses; for example, several immune regulators, including Toll-like receptor 7 (TLR7), are encoded on the X chromosome and have been shown to escape XCI in certain immune cell populations. The enhanced expression of such genes may promote stronger innate and adaptive immune responses in females. Thus, the interaction between estrogen signaling and X-linked gene regulation represents an additional layer of immune modulation that may contribute to the observed sex differences in asthma susceptibility and disease severity.

### 2.1 Receptor-specific and context-dependent effects of estrogen: ERα versus ERβ

Estrogen exerts context-dependent and receptor subtype-specific effects in asthma. Rather than acting as a uniformly pro-inflammatory mediator, estrogen produces opposing immunological outcomes depending on whether ERα or ERβ predominates. ERα generally drives immune activation, whereas ERβ exerts protective, anti-inflammatory, and bronchoprotective functions. ERβ-knockout female mice develop more severe allergen-induced airway pathology than ERα-knockout or wild-type controls, demonstrating that ERβ signaling constitutively restrains airway inflammation and remodeling *in vivo* (19). In human airway smooth muscle cells, ERα and ERβ agonists exert opposing effects on histamine-induced Ca²^+^ responses and contractility, with ERβ activation promoting relaxation even under IL-13-driven inflammatory conditions (20). Estrogen’s effects on ILC2 responses are similarly dose-dependent: supraphysiological E2 exacerbates allergen-driven airway inflammation, whereas follicular-phase concentrations suppress interleukin 33 (IL-33)-induced ILC2 cytokine production ex vivo, opposing outcomes reported within the same experimental program (21). In patients with asthma, serum E2 inversely correlates with circulating ILC2 numbers, a finding that directly contradicts a simple estrogen-amplifies-ILC2 model (22). These discrepancies probably reflect a non-monotonic, dose-dependent relationship in which physiological E2 concentrations suppress circulating ILC2 abundance while supraphysiological levels promote tissue ILC2 activation. This distinction underscores the need to interpret estrogen’s immunological effects in light of the specific receptor subtype, hormone concentration, and experimental context from which each finding derives. These context-dependent principles are particularly relevant when interpreting estrogen regulation of type 2 innate lymphoid cells (ILC2s), for which receptor-specific effects appear to vary across tissues and experimental settings. The balance between ERα and ERβ is dynamically regulated by the inflammatory microenvironment. Allergen challenge downregulates pulmonary ERβ expression, progressively shifting signaling toward ERα dominance and potentially establishing a self-amplifying inflammatory cycle that may underlie the greater severity and treatment refractoriness of asthma in adult females.

## 3 Pathogenesis of T2-high asthma

ILC2s and eosinophils are the major sources of type 2 cytokines in the airway, and they play a critical role in the initiation of allergic inflammation. Accumulating evidence indicates that estrogen signaling contributes to the expansion of these cell populations through the activation of ERα rather than ERβ, triggering the activation of intracellular pathways, such as those involving GATA binding protein 3 (GATA3), which promote the proliferation and survival of ILC2s and eosinophils, thereby increasing their accumulation in lung tissue (21, 23). Emerging evidence suggests that estrogen signaling influences ILC2 expansion and cytokine production in a tissue- and context-dependent manner. Although ERα has been implicated in regulating ILC2 responses in specific tissues, including the uterus, direct regulation of pulmonary ILC2s by ERα remains incompletely defined. In contrast, androgen signaling exerts an opposing regulatory effect, with some studies reporting that androgen receptor activation restrains the differentiation of ILC2 progenitors into mature ILC2s and limits their responsiveness to epithelial-derived IL-33 in mouse models (24). Sex hormones act as critical regulators of type 2 immune responses in the airway by modulating the expansion and functional activity of key effector populations such as ILC2s and eosinophils. Despite the success of biologic therapies that target eosinophilic inflammation, such as anti-interleukin 5 (IL-5) antibodies, a subset of patients will continue to experience persistent symptoms and recurrent exacerbation (25, 26), suggesting that additional Th2 effector cells may be contributing to disease progression.

Previous studies have extensively documented the role of estrogen in the regulation of type 2 immune responses, particularly through its effects on eosinophils and ILC2s, both of which are key drivers of airway inflammation in asthma (21, 22, 27). However, whether estrogen directly interacts with mast cells to modulate their activation and contribute to the exacerbation of asthma remains incompletely understood.

Mast cells are tissue-resident immune cells derived from hematopoietic progenitors that complete their maturation in peripheral tissues (28–30). In the airways, they are primarily localized within the submucosa and around smooth muscle tissue, allowing them to play a central role in the modulation of allergic inflammation and airway hyperresponsiveness. In females with airway sensitivity, allergen-specific immunoglobulin E (IgE) bound to the high-affinity Fc receptor for IgE (FcϵRI) becomes cross-linked upon allergen exposure, triggering rapid mast cell degranulation and the release of a broad spectrum of preformed mediators, including histamine, lipid mediators, proteases, and cytokines, which initiate the early phase of allergic inflammation and contribute to bronchoconstriction, mucus production, airway inflammation, and structural remodeling in asthma (31–33). Mast cell activation can occur through both IgE-dependent and IgE-independent mechanisms (34), and a growing body of evidence suggests that hormonal signaling can directly modulate mast cell activation and enhance their responsiveness while promoting mediator release through ERα, thereby amplifying inflammatory responses in the airway (35, 36). In addition to classical genomic signaling through ERα and ERβ, estrogen can also initiate rapid intracellular signaling through the activation of membrane-associated receptors such as GPER, potentially further amplifying FcϵRI-dependent responses (36–38). Notably, ERα and ERβ play opposing roles in mast cell function, consistent with the receptor-subtype dichotomy established in Section 2.1. Although ERα signaling has been associated with enhanced mast cell degranulation and activation threshold lowering, ERβ activation may exert restraining effects on mast cell responsiveness. This receptor-specific divergence means that the net effect of estrogen on airway mast cell activation depends on the prevailing ERα:ERβ ratio within resident airway mast cells, which may vary between disease states and individuals. Disease-driven downregulation of ERβ, as reported in allergen-challenged lung tissue, could therefore disinhibit mast cell responses, contributing to the amplified early-phase allergic reactions observed in females with established asthma (39). However, direct evidence for ERβ-dependent suppression of mast cell activation specifically within the asthmatic airway has not yet been established, and this mechanistic inference awaits dedicated experimental validation.

Mast cell function is also strongly influenced by the stem cell factor (SCF)–KIT signaling axis, as such cells constitutively express the receptor tyrosine kinase KIT, which is essential for mast cell development, survival, and tissue residency (40–42). The binding of SCF to KIT induces receptor dimerization and the activation of downstream signaling pathways that regulate mast cell proliferation, survival, and cytokine production. Estrogen signaling intersects with these intracellular pathways and may potentiate KIT-mediated responses, thereby enhancing mast cell persistence and lowering their activation threshold under inflammatory conditions (43, 44). KIT signaling has also been shown to be associated with the upregulation of adhesion-related genes that promote mast cell accumulation within airway smooth muscle bundles, a feature that has been linked to airway remodeling and greater disease severity in asthma (45–47). Estrogen may potentiate mast cell activation and mediator release through these converging mechanisms, thereby strengthening mast cell–airway cell interactions during the remodeling phase of asthma. Ultimately, persistent mast cell activation contributes to structural alterations of the airway wall, including epithelial cell shedding, goblet cell hyperplasia, hypertrophy of airway smooth muscle bundles, subepithelial fibrosis, abnormal extracellular matrix deposition, increased tissue vascularity, and basement membrane thickening.

## 4 Immune cells involved in severe non-type 2 asthma

### 4.1 T helper and regulatory T cells

In addition to their well-recognized role in mediating type 2 inflammation, increasing evidence suggests that non-type 2 immune pathways also contribute substantially to the exacerbation of asthma (48, 49), and T helper 17 cell (Th17)-mediated neutrophilic inflammation has emerged as a prominent underlying mechanism. Patients with this phenotype often exhibit a mixed inflammatory pattern characterized by elevated neutrophil counts in sputum, accompanied by enhanced corticosteroid resistance (50). Cohort studies have further reported that circulating Th17 cell counts are higher in female patients with severe asthma compared to that in male patients, raising interest in the role of sex hormones in the regulation of these responses (51, 52). Experimental evidence from ovariectomized and obese murine models has further implicated ERα signaling as a required driver of obesity-associated Th17-mediated airway inflammation (53). Recent studies have indicated that estrogen signaling may promote Th17 cell activity through ERα signaling, which upregulates interleukin 23 receptor (IL-23R) expression on Th17 cells and enhances their metabolic activity, including increased oxygen consumption and adenosine triphosphate production (54), thereby sustaining IL-17A production. This further amplifies inflammatory responses by stimulating airway epithelial cells, fibroblasts, and macrophages to produce a broad range of pro-inflammatory cytokines and chemokines, including interleukin 6 (IL-6), granulocyte colony stimulating factor (G-CSF), C-X-C motif chemokine ligand 1 (CXCL1), and C-X-C motif chemokine ligand 8 (CXCL8). These mediators promote the recruitment and activation of neutrophils at sites of airway inflammation, thereby sustaining neutrophilic inflammatory responses in the lung (48, 55). Consistent with this mechanism, ovariectomized mouse models of asthma exhibit markedly reduced Th17 cell responses and neutrophil infiltration in both lung tissue and peripheral blood (53). Although cross-sectional design precludes causal inference and cannot exclude confounding by corticosteroid use, body mass index, or disease duration, the consistent association between elevated Th17 responses and female sex across independent cohorts nonetheless lends plausibility to the experimental evidence linking ERα signaling to neutrophilic airway inflammation in women.

T2 and non-T2 inflammatory pathways are not mutually exclusive, adding further complexity to the situation. Mixed granulocytic endotypes, characterized by co-existing eosinophilic and neutrophilic inflammation, are increasingly recognized in severe adult female asthma. Cohort data indicate that this mixed endotype, which is more prevalent in women (56), cannot be accounted for by a strictly linear Th17 model. The pleiotropic effects of estrogen on both Th2 and Th17 pathways, promoting immune amplification across multiple axes simultaneously, may better explain this phenotypic overlap than polarization along any single-pathway axis.

Regulatory T cells (Tregs) also play a central role in maintaining immune tolerance and preventing excessive immune activation in the airway. Tregs suppress effector T-cell responses and limit inflammatory damage through the production of anti-inflammatory mediators such as interleukin 10 (IL-10) and transforming growth factor beta (TGF-β), as well as through cell-contact-dependent mechanisms (57). Estrogen can regulate the expression of the transcription factor forkhead box P3 (FOXP3), the master regulator required for Treg lineage commitment and functional stability, and ERα signaling has been reported to enhance FOXP3 expression and promote the expansion and functional stability of regulatory T cells under physiological conditions, thereby contributing to immune homeostasis (58, 59). However, Treg function is highly dependent on the surrounding inflammatory milieu. Exposure to elevated levels of pro-inflammatory cytokines such as IL-6, interleukin 1 beta (IL-1β), and IL-23 commonly observed in Th17-dominant inflammatory responses can cause Tregs to undergo functional reprogramming and exhibit phenotypic plasticity; these so-called “ex-Tregs” or Th17-like Tregs display reduced suppressive capacity and may even contribute to the amplification of local inflammatory responses (60), a process that may be further influenced by estrogen signaling, which can modulate the balance between Treg stability and Th17 differentiation. Through interactions with ERα-dependent signaling pathways and cytokine networks, estrogen can promote Th17 polarization while simultaneously altering the transcriptional stability of FOXP3 in Tregs, thereby disrupting the equilibrium between the Th17 and Treg compartments. Such an imbalance in the Th17/Treg axis has been increasingly recognized as a key immunological feature of severe or steroid-resistant asthma. Thus, by favoring Th17-driven inflammation and compromising Treg-mediated immune regulation, estrogen may contribute to the establishment of a pro-inflammatory airway microenvironment that enhances tissue inflammation, promotes neutrophilic infiltration, and ultimately exacerbates disease severity.

### 4.2 Macrophages

Macrophages are highly plastic innate immune cells that can adopt diverse functional phenotypes in response to local microenvironmental signals. Type 2-associated macrophage responses may contribute to both protective repair processes and pathological remodeling in the context of asthma. In lung tissue, macrophages comprise two populations of cells with distinct origins and functions, including tissue-resident alveolar macrophages, which arise from embryonic progenitors and play an important role in maintaining pulmonary homeostasis, and monocyte-derived macrophages that are recruited during inflammatory responses. These macrophage subsets dynamically respond to cytokines and environmental stimuli and play a key role in shaping the inflammatory landscape of the airway. Classically activated M1 macrophages are typically induced by interferon-γ and microbial signals and are characterized by the production of pro-inflammatory mediators such as IL-1β, tumor necrosis factor alpha (TNF-α), and IL-6. In contrast, alternatively activated M2 macrophages are driven by type 2 cytokines, including IL-4 and IL-13, and are generally associated with tissue repair, remodeling of the extracellular matrix, and the resolution of inflammation, with anti-inflammatory mediators such as IL-10 and TGF-β further supporting macrophage polarization toward an M2-like phenotype and promoting immune regulation and tissue homeostasis (61, 62).

Estrogen regulates macrophage polarization through receptor subtype-specific mechanisms that are fundamentally opposing. ERα signaling promotes alternative (M2) macrophage activation by enhancing IL-4Rα expression, augmenting IL-4-induced STAT6 phosphorylation, and cooperating with PPARγ to drive the expression of canonical M2 markers including arginase-1, YM1, and FIZZ1. Female mice exposed to allergens exhibit greater IL-4Rα expression and stronger M2 polarization than male controls, effects that are ERα-dependent (63, 64). In contrast, ERβ activation suppresses LPS-induced iNOS expression and NF-κB nuclear translocation in macrophages, thereby restraining M1 polarization. Critically, allergen challenge itself reduces pulmonary ERβ expression while leaving ERα levels unchanged, suggesting that the inflammatory microenvironment of established asthma progressively dismantles ERβ-mediated anti-inflammatory restraint and shifts the ERα:ERβ balance toward ERα dominance (65). This receptor-specific duality means that the balance between ERα-driven pro-inflammatory activation and ERβ-mediated immunoregulation in airway macrophages is a critical determinant of whether estrogen signaling amplifies or restrains innate immune responses under a given inflammatory condition. During innate immune activation, such as that associated with Toll-like receptor 4 (TLR4) activation induced by exposure to microbial products or environmental triggers, estrogen can modify intracellular signaling pathways and shift macrophage responses toward a pro-inflammatory state. For example, experimental studies have shown that estrogen may inhibit phosphoinositide 3-kinase (PI3K)/protein kinase B (Akt) signaling in activated macrophages, thereby enhancing the production of inflammatory cytokines, including IL-1β, TNF-α, and IL-6 (66). This context-dependent regulation indicates that estrogen can potentiate macrophage-mediated inflammatory responses to innate immune stimuli, increasing the production of pro-inflammatory cytokines and enhancing the recruitment of inflammatory cells to the airway. This dynamic may partly account for the mixed and treatment-refractory inflammatory phenotypes disproportionately observed in adult females.

Emerging evidence from immunometabolism studies highlights glycolytic reprogramming as an important but underappreciated dimension of macrophage inflammatory biology in asthma. Allergen-driven glycolytic reprogramming of airway macrophages through a TLR2-dependent mechanism, and macrophage-specific inhibition of glycolysis alters both airway homeostasis and allergen-induced T2 inflammatory responses *in vivo* (67). Mitochondrial reactive oxygen species represent a further convergence point: macrophage mitochondrial dysfunction generates reactive oxygen species that can prime NLRP3 inflammasome assembly, thereby linking immunometabolism reprogramming to the IL-1β–neutrophilic axis of non-T2 asthma (68). Notably, sex-stratified analyses of airway macrophage subpopulations at the single-cell level are currently lacking and understanding how estrogen signaling intersects with immunometabolism reprogramming in airway macrophages represents a research priority.

### 4.3 B cells

B cells play a central role in mediating humoral immunity through the production of antigen-specific antibodies and via the establishment of persistent immunological memory. In allergic airway diseases such as asthma, B cells are particularly important owing to their ability to generate IgE antibodies that drive mast cell and basophil activation. IL-4 is a key cytokine that promotes B-cell activation, differentiation, and immunoglobulin class-switch recombination toward IgE production. Estrogen can influence this process indirectly by modulating T-cell–derived cytokine production through ERα signaling, thereby enhancing IL-4–dependent humoral responses associated with allergic inflammation. Through this mechanism, estrogen may amplify T cell–B cell interactions within germinal centers and facilitate antibody class switching during allergic immune responses (69). Sex differences also influence B-cell function and the efficiency of humoral immunity, with estrogen signaling representing an important regulatory pathway. B cells express relatively high levels of ERβ,through which estrogen can modulate B-cell survival and differentiation. Estrogen has also been shown to enhance the expression of anti-apoptotic molecules such as B cell lymphoma 2 (BCL2) in autoreactive B cells, thereby influencing B-cell tolerance and survival (70). Furthermore, several genes involved in immunoglobulin class switching contain ERβ-binding sites, suggesting that estrogen signaling may directly regulate class-switch recombination. Thus, estrogen exposure can promote memory B cell generation while also enhancing antibody responses following antigen stimulation (71).

Beyond hormonal regulation, sex chromosomes also contribute to sex-specific differences in B-cell responses. XCI normally ensures dosage compensation of X-linked genes between males and females; however, disruption or incomplete maintenance of XCI can lead to the reactivation of genes on the inactive X chromosome. The established evidence that TLR7 escape from XCI amplifies innate immune signaling and germinal center formation derives predominantly from autoimmune disease contexts, particularly systemic lupus erythematosus, where B-cell-intrinsic TLR7 signaling plays a well-characterized pathogenic role. Whether analogous XCI escape in IgE-producing B cells contributes to the heightened IgE responses observed in female patients with asthma has not been directly demonstrated and should be regarded as a testable hypothesis (72). Human pDCs in which TLR7 escapes from XCI correlate with significantly higher IFNα/β output at the single-cell level, and XCI escape magnitude is substantially greater in lymphoid than myeloid lineages, providing a cell-type-specific rationale for investigating XCI escape in B cells and pDCs in the asthma-specific context (73, 74). Notably, estrogen appears to interact with this genetic background, as its effects on class-switched memory B-cell frequencies and antibody production have been shown to be the most prominent in individuals carrying two X chromosomes (69). Collectively, these findings suggest that both estrogen signaling and sex chromosome-linked epigenetic regulation cooperate to shape B-cell responses, ultimately contributing to sex-related differences in both humoral immunity and allergic disease susceptibility.

### 4.4 Tissue-resident memory T cells

The potential contribution of tissue-resident memory T (TRM) cells to asthma pathogenesis is an emerging and biologically compelling area of investigation. However, direct evidence specifically linking estrogen-mediated TRM regulation to asthma pathogenesis in human airways is currently limited.

T cells are central regulators of adaptive immunity and play an essential role in the establishment of long-term immunological memory. During the resolution phase of a primary immune response, most of the activated T cells undergo apoptosis to restore immune system homeostasis and prevent excessive tissue damage. This contraction phase is largely regulated by Fas-dependent apoptotic signaling and the balance between pro- and anti-apoptotic molecules, including members of the Bcl family, in cluster of differentiation 4-positive (CD4^+^) T cells. Estrogen can enhance anti-apoptotic signaling in T cells, thereby allowing a proportion of effector T cells to escape apoptosis and continue exerting effector functions for a prolonged period of time. In parallel, another subset of activated T cells undergoes differentiation into long-lived memory populations, with TRM cells representing a specialized subset that permanently resides in non-lymphoid tissues, including at mucosal sites such as in the respiratory tract (75–77). Unlike circulating memory T cells, TRM cells remain localized within tissues, allowing them to mount rapid and robust immune responses upon re-encountering an antigen. Upon activation, TRM cells can proliferate locally and rapidly produce effector cytokines, thereby contributing to the amplification of local immune responses and airway hyperresponsiveness (78, 79). Increasing evidence suggests that TRM cells may play an important role in persistent airway inflammation and steroid-refractory severe asthma. Whether estrogen signaling shapes the composition or function of these airway TRM populations in female patients has not been directly investigated. Experimental evidence from non-pulmonary systems demonstrates that the relationship between estradiol and CD4^+^ TRM formation is dose- and receptor-dependent and carries direct implications for asthma pathogenesis. E2 acts through ERα in hematopoietic cells to considerably amplify the clonal expansion of primary antigen-specific CD4^+^ T cells and selectively promote IFN-γ-producing Th1 development *in vivo*, an effect that is abrogated in ERα-deficient but not ERβ-deficient mice at physiological concentrations (80). At mucosal sites contiguous with the lower airway, E2 treatment following intranasal immunization significantly increases the frequency of CD4^+^ TRM cells in the upper respiratory tract and augments both Th1 and Th17 TRM subsets through an IL-17-dependent pathway. Critically, these E2-expanded TRM cells alone, in the absence of circulating T cells blocked by FTY720, are sufficient for complete mucosal protection upon rechallenge (81). At the opposite pole, ERβ signaling in CD4^+^ T cells promotes FOXP3^+^ Treg differentiation and peripheral tolerance. ERβ deficiency or downregulation in the inflamed mucosa shifts the balance toward persistent effector CD4^+^ T cell responses. In the context of asthma, these converging mechanisms suggest that ERα-driven expansion and mucosal retention of CD4^+^ TRM cells may represent a hormonally regulated mechanism amplifying the cytotoxic CD103^+^ CD4^+^ TRM populations recently identified as dominant and pathogenic in severe asthmatic airways (82). Whether E2 preferentially expands the Th2 TRM subset that drives recurrent eosinophilic exacerbations or the cytotoxic Th1/Th17 TRM subset associated with steroid-refractory disease depends on the hormonal concentration and local cytokine environment. This distinction maps directly onto the mixed inflammatory endotypes disproportionately represented in adult females with severe asthma. Direct validation of E2-driven airway TRM expansion in female patients with asthma through sex-stratified bronchoscope sampling with concurrent hormonal profiling remains essential.

## 5 Estrogen regulation of airway structural cells

Airway structural cells, including epithelial cells, airway smooth muscle cells, fibroblasts, and endothelial cells, play a central role in maintaining airway integrity and function. In asthma, dysregulation of these structural components contributes to airway remodeling, a key determinant of disease severity and persistent airflow limitation. In addition to regulating immune responses, estrogen may also directly influence the behavior of airway structural cells. Airway remodeling involves multiple coordinated processes, including epithelial injury, smooth muscle expansion, extracellular matrix deposition, and excessive mucus production. Emerging evidence suggests that estrogen signaling directly affects epithelial cell homeostasis. A recent preclinical study demonstrated that targeted knockdown of ERα in airway epithelial cells attenuated both ferroptosis and epithelial–mesenchymal transition in an eosinophilic asthma mouse model, suggesting that ERα-driven lipid peroxidation may compromise epithelial barrier integrity in asthmatic airways (14, 63). Emerging evidence provides mechanistic support for a link between ERα signaling and ferroptosis in asthmatic airway epithelium. Mechanistically, ERα promotes ferroptosis in the inflammatory airway epithelium by upregulating ACSL4, the enzyme that esterifies polyunsaturated fatty acids into membrane phospholipids to generate ferroptosis substrates, and through suppression of SLC7A11 transcription, reducing cystine import and thereby depleting the glutathione required for GPX4-mediated lipid hydroperoxide detoxification (83). This mechanistic framework is further supported by the observation that ERα expression in bronchial epithelial cells correlates with airway wall area, subepithelial fibrosis, and airway smooth muscle thickness in patients with asthma, and with the regulation of EGFR, a receptor that mediates ligand-independent ERα activation and IL-33 release from the epithelium (64).

Beyond epithelial cells, estrogen also modulates airway structural dynamics through additional pathways. Estrogen-responsive genes may further shape airway physiology; for instance, ORMDL3, a well-established asthma susceptibility gene, is regulated by estrogen and participates in sphingolipid metabolism, which is closely associated with airway hyperresponsiveness and inflammation (65). Estrogen signaling also affects airway smooth muscle behavior. Activation of ERβ has been shown to regulate cytoskeletal organization and focal adhesion turnover, facilitating airway smooth muscle cell migration and contributing to smooth muscle remodeling. In parallel, ERα signaling has been implicated in mucus hypersecretion through ligand-independent activation of epidermal growth factor receptor pathways (66).

In addition, emerging evidence suggests that estrogen may influence fibroblast activation and extracellular matrix remodeling, further contributing to subepithelial fibrosis and airway wall thickening. Estrogen-dependent signaling has also been implicated in vascular remodeling through its effects on endothelial cell function, potentially enhancing angiogenesis and inflammatory cell recruitment within the airway microenvironment.

**Figure 1:**
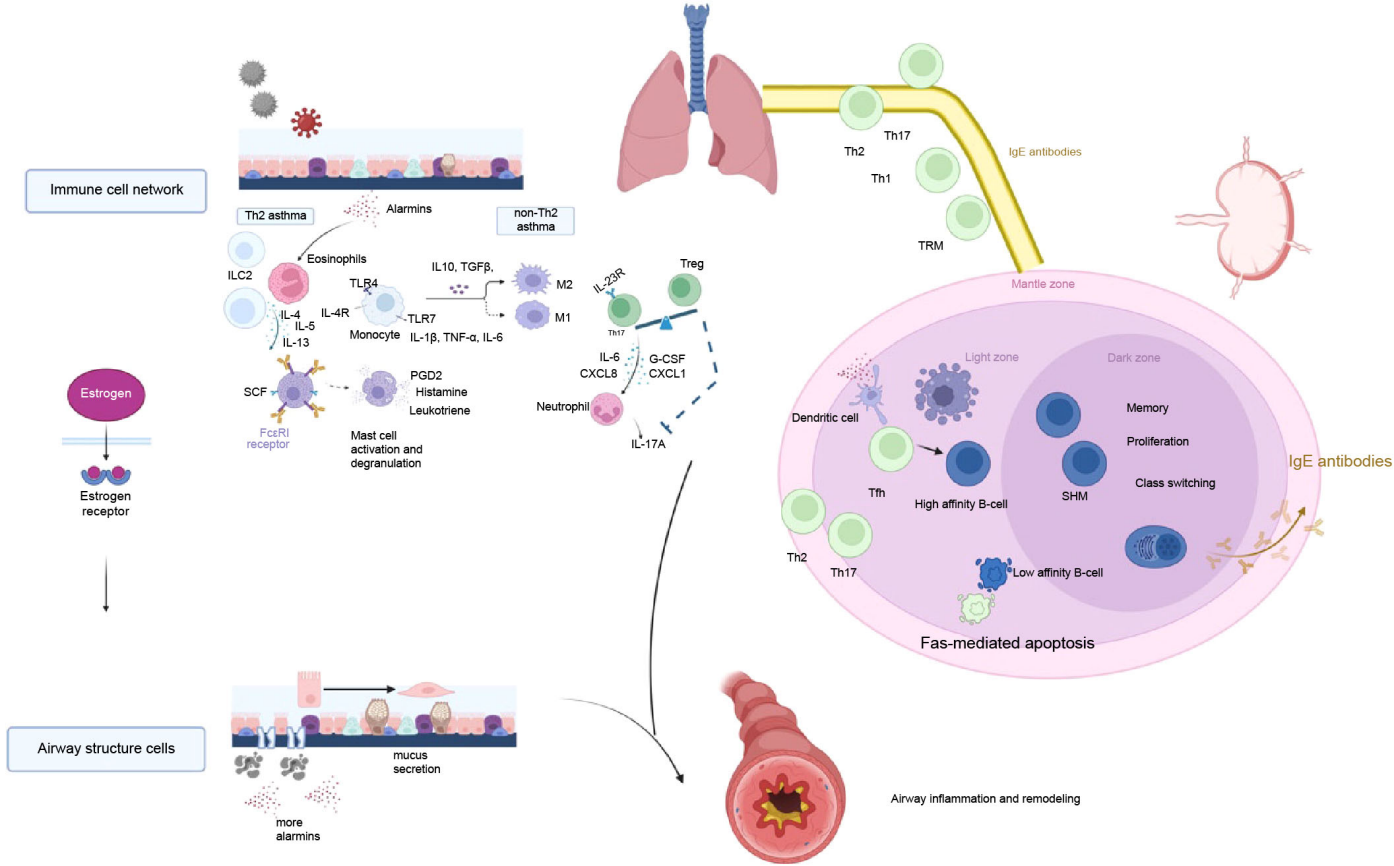
Estrogen-mediated regulation of immune cell responses and airway epithelial remodeling. ERα predominantly promotes pro-inflammatory responses by enhancing the activation of ILC2s, eosinophils, mast cells, Th17 cells, macrophages, and airway structural cells, thereby contributing to type 2 inflammation, neutrophilic inflammation, and airway remodeling. In contrast, ERβ generally exerts anti-inflammatory and bronchoprotective effects by restraining inflammatory signaling, preserving epithelial homeostasis, suppressing macrophage activation, and maintaining regulatory T-cell function and immune homeostasis. The overall immunological outcome is therefore determined by the dynamic balance between ERα- and ERβ-mediated signaling within both immune and airway structural cell populations. CXCL1, C-X-C motif chemokine ligand 1; CXCL8, C-X-C motif chemokine ligand 8; IgE, immunoglobulin E; IL-1β, interleukin 1 beta; IL-4, interleukin 4; IL-4R, interleukin 4 receptor; IL-5, interleukin 5; IL-6, interleukin 6; IL-10, interleukin 10; IL-13, interleukin 13; IL-17A, interleukin 17A; ILC2, group 2 innate lymphoid cell; PGD2, prostaglandin D2; SHM, somatic hypermutation; TGF-β, transforming growth factor beta; Th1, T helper 1; Th2, T helper 2; Th17, T helper 17; TLR4, Toll-like receptor 4; TLR7, Toll-like receptor 7; TNF-α, tumor necrosis factor alpha; TRM, tissue-resident memory T cell.

Collectively, these findings indicate that estrogen not only amplifies inflammatory signaling in the asthmatic airway but also directly contributes to structural remodeling of the airway wall. Through coordinated effects on epithelial integrity, airway smooth muscle dynamics, extracellular matrix deposition, and vascular remodeling, estrogen may promote persistent airway dysfunction and contribute to the increased disease severity and clinical heterogeneity observed in female patients with asthma. The integrated effects of estrogen on immune and structural compartments are summarized in **Figure 1**.

## 6 Asthma prevalence exhibits a sex switch across the lifespan

**Figure 2:**
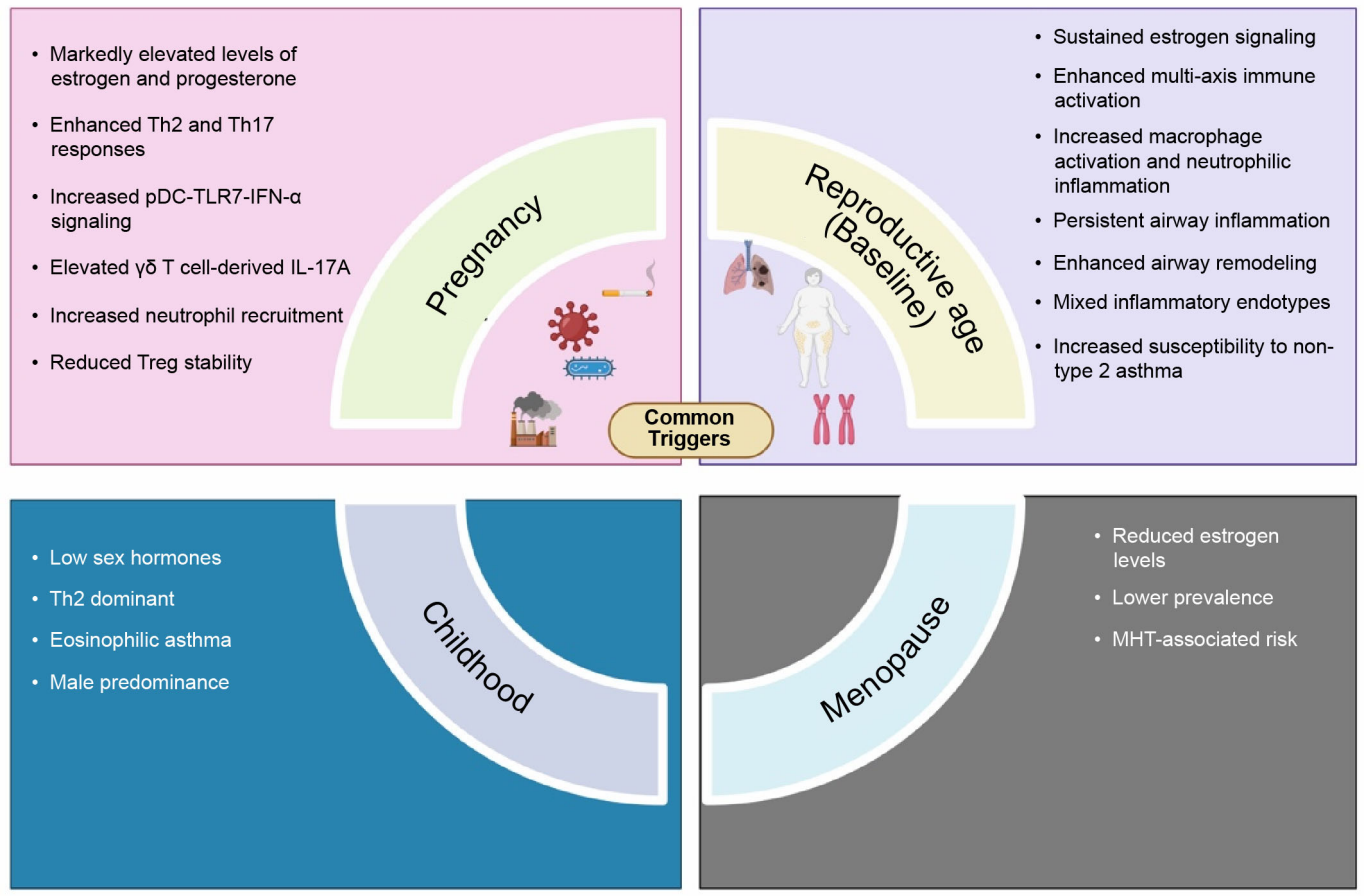
Life course-based overview of asthma risk, prevalence, and associated pathogenic mechanisms. IL-17A, interleukin 17A; MHT, menopausal hormone therapy; Th2, T helper 2; Th17, T helper 17; pDC–TLR7–IFN-α, plasmacytoid dendritic cell–Toll-like receptor 7–interferon alpha.

Pregnancy represents a unique physiological state that is characterized by profound fluctuations in hormone levels, particularly elevated levels of estrogen and progesterone; therefore, it provides a natural model to examine the hormone-driven modulation of asthma. Clinical observations have suggested that asthma control during pregnancy is highly variable, with approximately one-third of patients experiencing worsening symptoms (84). Beyond respiratory symptoms, maternal asthma is linked to adverse obstetric and neonatal outcomes, including pregnancy-related complications, an increased likelihood of spontaneous preterm birth, higher rates of neonatal hospitalization, and increased perinatal mortality (85). These findings suggest that hormone-mediated immune modulation not only affects asthma heterogeneity but is also associated with broader systemic and clinical consequences (**Figure 2**).

The epidemiological evidence regarding the relationship between menopause and asthma remains inconsistent. While some studies have reported no significant association between menopausal status and the prevalence or incidence of asthma, except among women undergoing menopausal hormone therapy (86), experimental studies have provided mechanistic insights. For example, in ovariectomized mouse models that mimic postmenopausal hormonal decline, allergen re-exposure has been shown to exacerbate airway inflammation, suggesting that the loss of estrogen may result in immune dysregulation and an increased susceptibility to inflammatory responses under specific conditions (87, 88). Data from other studies suggest that specific conditions, such as surgical menopause, may be associated with an increased risk of asthma. In contrast, in another study, no clear change in the incidence of asthma was observed in normal-weight women undergoing natural menopause (89). Given the contradictions, these findings should be interpreted with caution. It is also important to note that the clinical studies conducted to date have been limited by relatively small sample sizes, heterogeneity in the definition of menopausal status, and variability in key confounding factors, including age, obesity status, and hormone therapy use. Furthermore, current clinical assessments may not fully capture the relationship between menopause and asthma exacerbation or disease progression (86, 90). Consequently, the broader relationship between menopause and asthma remains complex and incompletely understood, and further prospective cohort studies and mechanistic investigations are required to clarify the associations.

In global studies, comorbidity or multimorbidity has been frequently observed in adults with severe asthma and has been shown to be associated with poorer clinical outcomes. Notably, epidemiological evidence suggests that sex-related differences across puberty are more pronounced in non-IgE-associated respiratory diseases; this indicates that other mechanisms beyond classical type 2 immunity may play a dominant role in driving disease progression and the development of related complications (91–95).

Non-type 2 asthma is commonly associated with viral infection, obesity, and exposure to environmental contaminants such as particulate matter, all of which collectively contribute to neutrophilic and biomarker-poor inflammation. Notably, these factors intersect with sex-dependent immune regulation and may disproportionately affect females. In addition to promoting systemic inflammation and oxidative stress within airway tissues, obesity heightens the susceptibility to environmental pollutant-induced injury. Female patients, particularly in late-onset asthma, often exhibit a more severe phenotype that is characterized by reduced eosinophilic inflammation accompanied by increased metabolic and oxidative dysregulation, which can be alleviated by body mass index reductions (96). Viral infection represents another critical driver of disease exacerbation and hospitalization. In females, heightened antiviral immunity, characterized by an increase in the number of IL-17A-producing cells, such as γδ T cells, accompanied by enhanced type I interferon production by plasmacytoid dendritic cells via TLR7 signaling, may promote excessive innate immune activation and neutrophilic inflammation (97). Among respiratory comorbidities, chronic rhinosinusitis and bronchiectasis are particularly relevant to the heterogeneity of asthma.

Several inflammatory pathways beyond the classical T2/Th17 axes are emerging as key contributors to severe and treatment-refractory asthma, and their intersection with sex hormone-dependent immune regulation warrants discussion. NLRP3 inflammasome activation in airway macrophages and neutrophils generates caspase-1-dependent IL-1β and IL-18, which promote neutrophil survival, amplify corticosteroid resistance, and sustain steroid-refractory airway inflammation (98). ERα signaling promotes NLRP3 transcription through direct binding to estrogen response elements on the *NLRP3* gene promoter, with selective ERα agonism markedly upregulating activation-associated gene expression across inflammatory disease models (99). In the asthmatic airway specifically, allergen challenge increases NLRP3 inflammasome activation while reducing pulmonary ERβ expression, and exogenous E2 suppresses this NLRP3 upregulation in an ERβ-dependent manner. In contrast, ERβ activation suppresses NLRP3 activity in lung macrophages, and allergen challenge reduces ERβ expression in lung tissue, potentially removing this protective brake and permitting NLRP3-driven neutrophilic inflammation to escalate (65, 100). Together, these findings suggest that the shift toward ERα dominance driven by disease-induced ERβ downregulation does not merely remove an anti-inflammatory brake but actively licenses NLRP3-driven IL-1β production, potentially explaining why neutrophilic, steroid-resistant airway inflammation escalates preferentially in females with established severe asthma.

Neutrophil extracellular traps (NETs) are extracellular scaffolds of DNA decorated with histones, neutrophil elastase, and myeloperoxidase. NETs have been detected in the sputum and bronchoalveolar lavage fluid of patients with severe asthma and correlate with disease severity (101). Beyond their direct cytotoxic effects on airway epithelium, NETs stimulate dendritic cells to produce IFN-α, IL-6, and IL-12/p70, driving Th1/Th17 polarization and amplifying corticosteroid resistance (102). The intersection of sex hormones with NETosis in the asthmatic airway has not been directly studied. However, given the female predominance of severe neutrophilic asthma and the ERα-driven promotion of neutrophil activation and survival described above, the question of whether estrogen signaling amplifies NET formation, and whether this contributes to the greater airway structural damage and steroid resistance observed in female patients, represents an interesting hypothesis for future investigation.

The gut microbiome influences systemic estrogen bioavailability through bacterial β-glucuronidase activity, which represents the enzymatic capacity of certain gut microbiota to deconjugate estrogen glucuronides in the intestinal lumen, enabling reabsorption of free estrogen (103). The relationship between gut microbiome dysbiosis and asthma is not sex neutral. Allergen challenge induces fundamentally different gut microbial responses in males and females. In a murine HDM model, alpha diversity decreased selectively in females while increasing in males, and Bacteroidetes, a bacterial phylum that exhibits pro-inflammatory properties, was more abundant in the female gut microbiota at baseline and following allergen exposure (104). These sex-specific microbial shifts coincided with greater neutrophilia, lymphocytosis, and histopathological severity in females, suggesting that the female gut microbiome may constitute an amplifying substrate for allergic airway inflammation. The systemic consequences extend beyond the lung: chronic allergic lung inflammation driven by house dust mite sensitization in female mice disrupts gut microbiota β-diversity and is associated with depression-like behavioral changes through the gut-brain axis (105). These observations integrate with the estrobolome concept to suggest a self-reinforcing hormonal-microbial circuit specific to females. The gut microbiome regulates systemic estrogen bioavailability through bacterial β-glucuronidase activity, by which estrobolome bacteria deconjugate estrogen glucuronides in the intestinal lumen and enable the reabsorption of free estrogen. In females with asthma-associated gut dysbiosis, particularly in the obesity-asthma phenotype, reduced microbial diversity and shifts in phylum composition may alter β-glucuronidase activity and thereby modify circulating estrogen levels independently of ovarian function. The resulting dysregulation of systemic estrogen could in turn amplify ERα-dependent airway inflammatory responses, contributing to the greater metabolic and oxidative dysregulation disproportionately observed in adult females with late-onset or obesity-associated asthma. Although the full mechanistic chain linking asthma-induced gut dysbiosis, estrobolome disruption, altered estrogen signaling, and airway immune amplification has not yet been prospectively characterized in humans, the convergent evidence from sex-specific microbial studies and estrogen receptor biology provides a compelling and testable framework for understanding why the gut–lung–hormone axis may function as a sex-specific amplifier of asthma severity.

Chronic rhinosinusitis is more prevalent in females than in males, whereas chronic rhinosinusitis with nasal polyps is more prevalent in males but tends to be more severe in female patients, with a stronger association with asthma and severe phenotypes such as Aspirin-exacerbated respiratory disease (106, 107). In addition, bronchiectasis frequently coexists with overlapping asthma–chronic rhinosinusitis and is associated with increased disease severity, exacerbation risk, and impaired pulmonary function (108). Notably, these comorbidities define a subgroup of patients with more complex and severe airway inflammation, which further contributes to the heterogeneity of asthma, particularly in females.

Building upon the multifaceted effects estrogen exerts on immune and structural cells, an important question arises as to how these mechanisms translate into sex-specific differences in asthma phenotypes. Epidemiologically, the prevalence of asthma is higher in males than in females during childhood, during which time the disease is predominantly characterized by type 2 inflammation; however, there is a shift toward female predominance and increased disease severity after puberty. Notably, adult female patients display greater clinical heterogeneity, with a higher prevalence of non-type 2 asthma. This transition cannot be explained by a simple enhancement of Th2-mediated immunity; instead, the pleiotropic effects of estrogen on multiple immune pathways, including the concurrent promotion of Th2 and Th17 responses, neutrophil recruitment, macrophage activation, and disruption of Treg stability, suggest that immune amplification and dysregulation play a broader role in regulating such differences. These coordinated effects expand the inflammatory repertoire rather than polarizing it toward a single axis. Consequently, post-pubertal estrogen exposure may lower the threshold for immune deviation, thereby increasing the susceptibility to mixed or non-type 2 inflammatory patterns, particularly under additional pathological conditions. This provides a mechanistic framework linking sex hormone-mediated immune modulation to the increased severity and heterogeneity of asthma observed in adult females.

## 7 Discussion

A key theme emerging from this review is that estrogen’s immunological effects are neither uniform nor unidirectional. ERα and ERβ frequently exert opposing effects on the same cell types, with ERα predominantly promoting immune activation in the context of established airway inflammation and ERβ providing protective anti-inflammatory counter regulation. Disease progression in severe asthma is associated with ERβ downregulation in lung tissue, suggesting that the ERα:ERβ balance shifts toward ERα dominance as disease severity increases, potentially creating a self-amplifying cycle in which inflammatory escalation further removes the ERβ-dependent braking mechanism. This receptor-balance model can reconcile many of the apparent contradictions in the estrogen-asthma conclusions. Rather than driving a single inflammatory pathway, estrogen promotes immune amplification and dysregulation through its effects on multiple immune cell populations and airway structural cells, expanding the inflammatory repertoire and lowering the threshold for immune deviation. Consequently, females, particularly during the reproductive period and pregnancy, are more likely to develop severe and non-type 2 asthma characterized by neutrophilic inflammation and greater disease heterogeneity, with common triggers such as obesity, viral infection, environmental contaminant exposure, and respiratory comorbidities further shaping disease expression. Although these factors are shared across life stages, their impact depends on the underlying hormonal context, which can lead to distinct inflammatory patterns under different physiological conditions. Collectively, these observations support a model in which asthma heterogeneity arises from the interaction between shared environmental triggers and hormone-dependent immune regulation, highlighting sex and life stages as central determinants of disease phenotype and severity.

These insights have implications for the clinical management of asthma. The predominance of non-type 2 inflammation and increased disease heterogeneity observed in certain female populations may contribute to reduced responsiveness to corticosteroid therapy and limit the efficacy of type 2-targeted biologics. Patient stratification can be improved by basing it on hormonal status, life stage, and inflammatory phenotype. Emerging therapies targeting non-type 2 pathways, including NLRP3 inhibitors (e.g., MCC950), anti-IL-1β agents, and PAD4 inhibitors targeting NETosis, may be particularly relevant for female patients with severe neutrophilic asthma, and their efficacy may be modulated by ERα:ERβ balance and hormonal context. Future clinical trials in severe asthma should aim to routinely stratify participants by sex, hormonal status, and inflammatory endotype to facilitate the identification of sex-specific treatment responses.

Despite the growing body of evidence linking estrogen to asthma pathogenesis, the mechanisms underlying sex-related disease heterogeneity remain incompletely understood. Future studies should move beyond a hormone-centered framework and consider asthma to be the result of a coordinated system of regulation involving hormonal signaling, sex chromosome-linked mechanisms, and epigenetic programming. Interactions between estrogen signaling and XCI, including escape from X-linked gene silencing, may represent an additional layer of immune regulation that has yet to be fully elucidated.

The origins of sex differences in early life also remain unclear. For example, the higher prevalence of asthma observed in boys before puberty, when circulating sex hormone levels are low, suggests that developmental immune programming, lung maturation, or sex chromosome-dependent mechanisms contribute to a matter that is independent of hormonal influences. How these early-life factors interact with hormonal regulation later in life remains unknown. Hormonal fluctuations occurring during pregnancy and menopause in women further complicate this landscape, reshaping immune responses and disease phenotypes in ways that are not yet fully understood, particularly in relation to non-type 2 inflammation and severe asthma. Longitudinal studies integrating clinical data with immune profiling across different life stages will be essential to clarify these dynamic changes over time. Emerging technologies, including single-cell multi-omics, spatial transcriptomics, and epigenetic profiling, are expected to provide new opportunities to dissect these complex interactions, and applying these approaches could enable the identification of sex-specific biomarkers and support the development of more precise, context-dependent therapeutic strategies.

A more complete understanding of the ways in which hormonal context, genetic regulation, and environmental triggers converge to shape asthma heterogeneity will be critical for advancing precision medicine and improving clinical outcomes in female patients.
